# Outcomes Following Surgery for Pancreatic Neuro-Endocrine Tumours: A Single-Centre Experience

**DOI:** 10.3390/clinpract15110202

**Published:** 2025-10-30

**Authors:** Mina Fouad, Sayed Ali Almahari, Abed Moeti Zaitoun, Prithvirao Sonoo, Sepand Malek, Karim Sourial, Dhanny Gomez

**Affiliations:** 1 Department of HPB Surgery, Nottingham University Hospitals NHS Trust, Queen’s Medical Centre, Derby Road, Nottingham NG7 2UH, UK; m.fouad@nhs.net (M.F.); prithvirao.sonoo@nhs.net (P.S.); sepand.malek1@nhs.net (S.M.); karim.sourial@nhs.net (K.S.); dhanny.gomez1@nhs.net (D.G.); 2 Department of Cellular Pathology, Nottingham University Hospitals NHS Trust, Nottingham NG7 2UH, UK; sayedali.almahari@nhs.net; 3 Nottingham Digestive Diseases Centre, Division of Translational Medical Sciences, School of Medicine, University of Nottingham, Queen’s Medical Centre, Derby Road, Nottingham NG7 2UH, UK

**Keywords:** pancreas, neuroendocrine tumours, recurrence, surgery, prognosis

## Abstract

Aims: The purpose of this study was to evaluate survival outcomes and recurrence patterns following curative-intent resection of pancreatic neuroendocrine tumours (PNETs) at a UK tertiary centre. The secondary aims included identifying prognostic clinicopathological factors that influenced survival. Methods: Patients undergoing curative-intent surgical resection for PNETs between August 2010 and March 2024 were retrospectively reviewed. The data collated included demographics, histopathology, recurrence, and survival outcomes. Results: Eighty-six patients were included, with a median age of 61.5 years (IQR: 50–71) and an equal sex distribution. Most tumours were solitary (88.4%) and located in the pancreatic tail (57%), with distal pancreatectomy performed in 75% of cases. The median tumour size was 25 mm (IQR: 13–40). Lymph node metastases were observed in 23.3% of patients, and R0 resection was achieved in 67%. Most of the PNETs resected were WHO grade 1 tumours (65.1%), followed by grade 2 tumours (26.7%). Postoperative morbidity occurred in 37.2% of cases, while the 30-day postoperative mortality rate was 1.5%. Recurrence was observed in 13.95% of patients, with a median time to recurrence of 36.3 months. The 5-year overall survival (OS) was 83.0%, with a median OS and disease-free survival (DFS) of 143.3 months and 147.0 months, respectively. Multivariable analysis revealed that poorer DFS was associated with larger tumours (*p* = 0.009), higher tumour grade (*p* = 0.006), male sex (*p* = 0.039), vascular invasion (*p* = 0.003), perineural invasion (*p* = 0.042) and lymph node metastases (*p* = 0.015). OS was significantly influenced by the Charlson Comorbidity Index (*p* < 0.001) and tumour grade (*p* = 0.025). Conclusions: PNETs are associated with excellent long-term survival following curative-intent resection. However, adverse pathological features are linked to an increased risk of recurrence and a poorer prognosis.

## 1. Introduction

Pancreatic neuroendocrine tumours (PNETs) are uncommon neoplasms accounting for approximately 1–2% of all pancreatic malignancies and fewer than 10% of neuroendocrine tumours (NETs) overall [1,2]. Most PNETs arise sporadically, while approximately 10–30% of PNETs are associated with hereditary syndromes, including Multiple Endocrine Neoplasia type-1 (MEN-1), von Hippel–Lindau disease (VHL), neurofibromatosis type-1 (NF-1) and tuberous sclerosis complex (TSC) [3].

Approximately 15–40% of PNETs are functionally active, secreting bioactive peptides that give rise to distinct clinical syndromes such as insulinoma or gastrinoma [4,5]. The biological behaviour of PNETs tends to be broad-spectrum, ranging from indolent, well-differentiated neoplasms to highly aggressive tumours [6]. Tumour grade for PNETs is determined by mitotic count and the Ki–67 proliferation index, which is a critical prognostic marker and forms the basis of the current World Health Organization (WHO) classification system (grades G1–G3) [6,7].

With the increased usage of cross-sectional imaging in secondary care, this has led to the recent increase in incidence of early-stage, resectable PNETs. As a direct consequence, the number of pancreatic resections has increased, making PNETs the second most common indication for pancreatic surgery, after pancreatic ductal adenocarcinoma [8,9,10].

Surgical resection remains the only potentially curative treatment, with 5-year survival rates exceeding 75% for patients with well-differentiated, localised tumours. Nevertheless, disease recurrence occurs in approximately 10–15% of cases, most commonly as distant metastases. The long-term prognosis in this subgroup remains poorly defined [7]. Although several studies have investigated prognostic indicators in PNETs, further data is required to guide the individualised management of recurrent disease and surveillance programmes.

The aim of this study was to determine recurrence patterns and survival outcomes following curative-intent resection of PNETs at a tertiary centre. Secondary aims include identifying clinicopathological variables that influenced survival in this group of patients.

## 2. Methods

### 2.1. Study Design, Ethics Approval and Protocol

**Figure 1:**
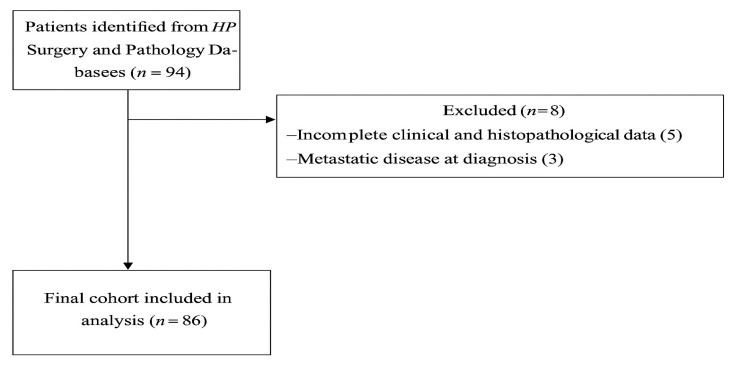
STROBE flowchart of patient selection for curative surgery in PNETs. This diagram illustrates the study cohort derivation from August 2010 to March 2024. Of 94 initially identified patients, 86 met the inclusion criteria (histologically confirmed PNETs, curative-intent resection, complete data). Exclusions: Five due to incomplete records and three with metastatic disease at diagnosis. The final cohort (*n* = 86) had a median follow-up of 143.3 months, with 82.6% surviving at last follow-up.

This was a retrospective study of patients who underwent curative-intent surgical resection for PNETs at Queen’s Medical Centre, Nottingham University Hospitals NHS Trust, Nottingham, United Kingdom. All patients who underwent pancreatic resections with curative intent between August 2010 and March 2024 were included. All patients were identified through the Hepato-Pancreato-Biliary (HPB) Surgery departmental database and the Cellular Pathology database. The study was conducted in accordance with the Declaration of Helsinki. Ethical review and approval were waived based on the UK National Health Service Research Authority Decision Tool (http://www.hra-decisiontools.org.uk/research/index.html, accessed on 29 March 2025) and confirmed by the Clinical Audit department at Nottingham University Hospitals NHS Trust (project ID number: 25-228C), Nottingham, as this study was not classed as Research by the National Health Service (NHS). Patient consent was waived since this study was classified as a service evaluation This study was reported according to STROBE guidelines (Figure 1) [11].

Inclusion criteria included consecutive adult patients (≥18 years) undergoing surgical resection for PNETs. Power calculation for sample size was not performed in this study. This study excluded all patients who underwent explorative surgery only, surgery for other pancreatic tumours and patients with metastatic disease at the time of diagnosis. In addition, patients with incomplete clinical and survival data were excluded.

### 2.2. Clinical Data

Patient data was obtained from the HPB Surgery departmental database, the Cellular Pathology database and hospital electronic medical records. Data accuracy and completeness were independently verified by two investigators (MF and AZ).

Demographics, pre-operative staging, surgery and postoperative outcomes (morbidity and 30-day mortality) and histologic analysis were recorded and analysed in the collective database. All patients were staged with computed tomography (CT) of the chest, abdomen and pelvis. An octreotide scan was routinely performed if the CT scan suggested PNETs or there was histological confirmation from endoscopic ultrasound biopsies. Magnetic resonance imaging (MRI) of the liver was performed if suspicious lesions were identified on the CT or Octreotide scans. ^18^ FDG-Positron emission tomography (PET) was used selectively. Liver metastases found on pre-operative staging were a contraindication for resection.

Pathological analysis included tumour size (maximum dimension), number of lesions, UICC (8th edition) TNM classification of malignant tumours, tumour grade (G1–G3), and Ki–67 proliferation index. Resection margin status was defined as a clear margin (R0) if no tumour was present at or within 1 mm of the surgical margin. The presence of vascular and perineural invasion was noted. Additional data included tumour functionality (functioning versus non-functioning), associated hereditary syndromes (e.g., MEN1, VHL), and any concurrent pathological diagnoses.

### 2.3. Outcome Variables

Postoperative complications were classified according to the Clavien–Dindo (CD) classification system, with grade > IIIa considered major morbidity [12]. Patients were followed up clinically at six-monthly intervals for the first three years postoperatively, and annually thereafter. Surveillance CT was performed annually. Change in clinical status led to earlier clinical review and potentially repeat CT imaging.

Disease-free survival (DFS) was defined as the time from surgery to the first documented disease recurrence on imaging. Overall survival (OS) was defined as the time interval between the date of surgery and date of death or most recent date of follow-up if the patient was still alive. Overall survival following recurrence (OSR) was defined as the time interval between the date of recurrence diagnosis and date of death or most recent date of follow-up if the patient was still alive. Thirty-day mortalities were excluded from survival analysis.

## 3. Statistical Analysis

Categorical variables were summarised as frequencies and percentages. Comparisons between categorical groups were performed using the chi-squared test or Fisher’s exact test, as appropriate. Continuous variables were reported as medians with interquartile ranges (IQR).

Survival outcomes, including OS, DFS and post-recurrence OSR, were estimated using the Kaplan–Meier method, with differences assessed using the log-rank test.

Univariable analysis was performed to identify variables that influenced disease-free and overall survival. A two-sided *p* < 0.05 was considered significant. Cox proportional hazards regression analysis (forward stepwise regression) was used for multivariable analysis to further evaluate variables significant on univariable analysis, with coefficients reported as hazard ratios (HRs) and corresponding 95% CIs. Variables with *p* < 0.05 in univariable analysis were included in the final multivariable models. All statistical analyses were conducted using SPSS Statistics for Windows^TM^, Version 25.0 (IBM Corp., Armonk, NY, USA).

## 4. Results

### 4.1. Patients’ Demographics and Pathological Features

Of the 94 patients initially identified, 86 patients who underwent pancreatic resections with curative intent were included in the final analysis (Figure 1). Eight patients were excluded due to incomplete data (*n* = 5) or the presence of metastatic disease at diagnosis (*n* = 3). The median age was 61.5 years (IQR: 50–71), with equal gender distribution. The median Charlson Comorbidity Index (CCI) was 4 (IQR: 3–5). The overall median follow-up was 61.5 months (IQR: 27.2–97.3), during which 12 (13.9%) patients developed recurrence, and 71 (82.6%) patients were alive at last follow-up.

### 4.2. Surgery and Postoperative Morbidity and Mortality

Distal pancreatectomy was the most frequently performed operation (75.6%, *n* = 65, Table 1).

Overall postoperative morbidity was reported in 32 (37.2%) patients. CD *>* IIIa was observed in five (15.6%) patients. The most common complications were intra-abdominal collections (*n* = 7, 8.1%), ileus (*n* = 6, 6.9%), and chest infections (*n* = 4, 4.7%). Pancreatic leaks were observed in four (4.6%) patients, as were cases of pancreatitis and postoperative bleeding (each 2.3%). Thirty-day mortality was 3.1% (*n* = 1).

### 4.3. Histopathological Analysis

The pancreatic tail was the most common tumour site (*n* = 49, 57.0%), followed by the head (*n* = 15, 17.4%,) and body (*n* = 11, 12.8%). Most tumours were solitary (*n* = 76, 88.4%). The median tumour size was 25 mm (IQR: 13–40 mm), and T1 tumours accounted for 40.7% (*n* = 35) of cases. N1 and N2 disease were reported in 22.1% (*n* = 19) and 1.2% (*n* = 1), respectively.

Most tumours were WHO grade I (*n* = 56, 65.1%), with a Ki–67 index <3%. R0 resection was achieved in 77.9% (*n* = 67) of patients. Perineural and vascular invasion were present in 13.9% (*n* = 12) and 29.1% (*n* = 25), respectively. Functioning tumours were identified in 24 (27.9%) patients, most commonly insulinomas (*n* = 10, 41.7%). Genetic syndromes were present in 9.3% (*n* = 8) of patients.

### 4.4. Recurrence

Disease recurrence occurred in 12 patients (13.9%), with a median time to recurrence of 36.3 months (IQR: 8.0–53.8). The median age at recurrence was 56.1 years (IQR: 41.3–71.0), and most affected patients were male (*n* = 9, 75.0%; male-to-female ratio 3:1). The median Charlson Comorbidity Index was 5.75 (IQR: 3.0–8.25). Distal pancreatectomy was the most common initial procedure (*n* = 6, 50.0%).

At the time of primary resection, T2 tumours were most common (*n* = 5, 41.7%), while T1 tumours were least frequent (*n* = 1, 8.3%). Nodal metastases (N1) were identified in 41.7% (*n* = 5). The majority were unifocal tumours (*n* = 11, 91.7%) with a median tumour size of 30.3 mm (IQR: 25.0–35.0). Vascular invasion was present in 66.7% (*n* = 8), perineural invasion in 33.3% (*n* = 4), and positive resection margins (R1) in 25.0% (*n* = 3). High-grade tumours (G3, Ki–67 >20%) were seen in 41.7% (*n* = 5), low-grade (G1, Ki–67 <3%) in 33.3% (*n* = 4), and intermediate-grade (G2, Ki–67 3–20%) in 25.0% (*n* = 3).

Co-existing pathology was noted in 25.0% (*n* = 3), including IPMN (*n* = 2, 16.7%), pancreatic adenocarcinoma (*n* = 1, 8.3%), and genetic syndromes, namely von Hippel–Lindau disease (*n* = 2, 16.7%), which showed a statistically significant association with recurrence (Fisher’s exact test, *p* = 0.012). Most tumours were non-functioning (*n* = 11, 91.7%), with a single insulinoma (*n* = 1, 8.3%).

**Figure 2:**
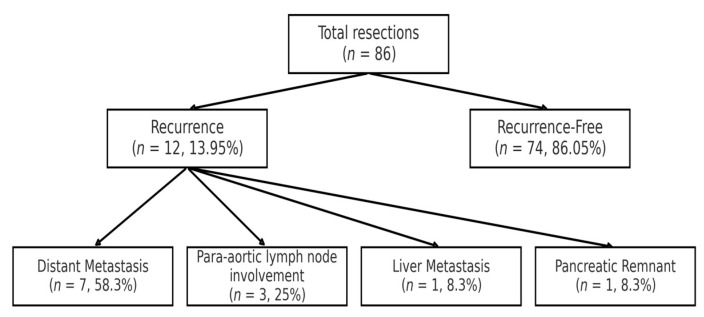
Patterns of recurrence in patients with PNETs. Recurrence occurred in 13.95% (12/86) of patients, predominantly as distant metastases (58.3% liver/lung, 25% para-aortic nodes). Median time to recurrence was 36.3 months (IQR: 8–53.75). High-risk features in recurrent cases were as follows: 66.7% had vascular invasion, 41.7% were grade G3 (Ki–67 >20%), and 41.7% had nodal involvement (N1). Late recurrences (>5 years) correlated with VHL syndrome (*p* = 0.03) and lower Ki–67 (<0.25%, *p* = 0.01).

Patterns of recurrence (Figure 2) included combined liver and lung metastases (*n* = 7, 58.3%), para-aortic lymph node involvement (*n* = 3, 25.0%) and isolated liver metastasis treated with surgical resection (*n* = 1, 8.3%). Local recurrence in the pancreatic remnant occurred in one patient (8.3%) and was managed with completion pancreatectomy. Most patients (*n* = 8, 66.7%) received adjuvant chemotherapy, with additional treatments including completion pancreatectomy (*n* = 1, 8.3%), immunotherapy (*n* = 1, 8.3%) and surgical resection of isolated liver metastases (*n* = 1, 8.3%). Table 2 summarises the histopathological data from the primary tumour specimens at the initial surgical resection.

### 4.5. Survival Outcomes

During the follow-up period, 15 (17.4%) patients died due to metastatic PNET (*n* = 7, 46.7%), unrelated cardiovascular or other medical causes (*n* = 6, 40%), and co-existing metastatic breast cancer (*n* = 2, 13.3%).

**Figure 3:**
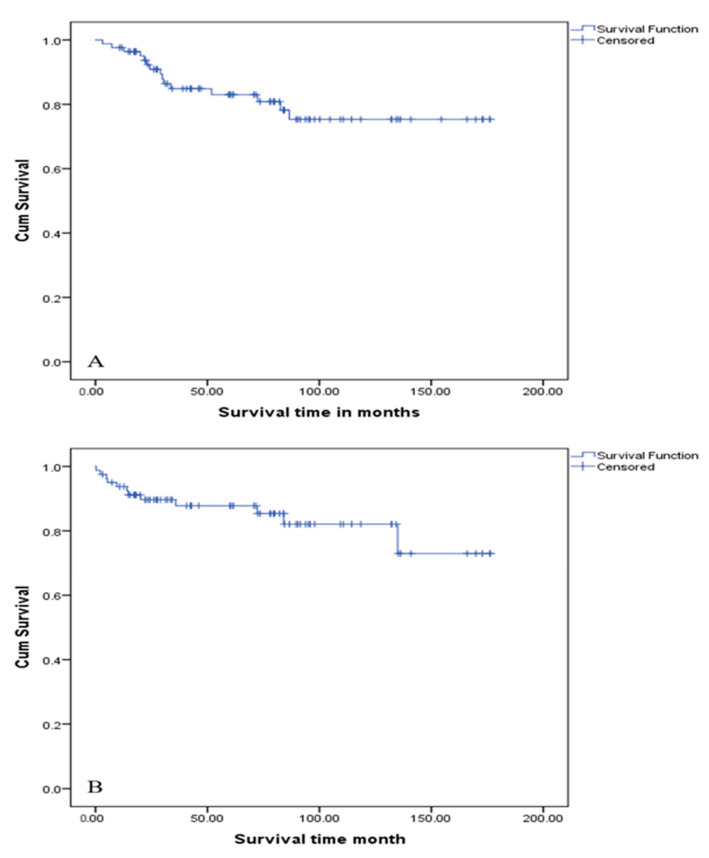
(**A**) Kaplan–Meier survival curve for overall survival of the entire cohort. (**B**) Kaplan–Meier survival curve for disease-free survival. The cohort (*n* = 86) demonstrated excellent long-term survival: 1-year OS 97.6% (95% CI: 94.0–100%) and 5-year OS 83.0% (95% CI: 74.6–92.4%). Median OS was 143.3 months (95% CI: 128.5–158.1). Multivariate analysis identified the Charlson Comorbidity Index (CCI; *p* < 0.001) and tumour grade (*p* = 0.025) as independent predictors of survival. Mortality was driven by metastatic PNET (46.7%) or comorbidities (40%). Median DFS was 147.0 months (95% CI: 131.8–162.2). Shorter DFS correlated with larger tumour size (HR 1.013, *p* = 0.009), vascular invasion (HR 6.35, *p* = 0.003), nodal metastases (HR 4.53, *p* = 0.015) and male sex (HR 4.03, *p* = 0.039). Late recurrences (> 5 years) had superior survival (0% mortality vs. 77.8% in early recurrences, *p* = 0.02).

The median DFS was 147.0 (IQR: 131.8–162.2) months, and the 5-year DFS was 82.8%. Multivariable analysis identified male gender (*p* = 0.039), large tumour size (*p* = 0.009), high-grade tumours (*p* = 0.006), vascular invasion (*p* = 0.003), perineural invasion (*p* = 0.042) and the presence of nodal disease (*p* = 0.015) as independent predictors of poorer DFS (Figure 3/Table 3).

The median OS for the cohort was 143.3 months (IQR: 128.5–158.1) and the 5-year OS was 83.0% (Figure 3/Table 4). Large tumour size (*p* = 0.007), poor CCI (*p* < 0.001), high Ki–67 proliferation index (*p* = 0.02) and high-grade tumours (*p* = 0.025) were independent prognostic factors on multivariable analysis. Functional pancreatic tumours had a higher 5-year survival rate (83.3%) compared to non-functional tumours (66.7%); however, that was statistically insignificant (*p* = 0.48). Perioperative deaths were excluded from the OS analysis.

**Figure 4:**
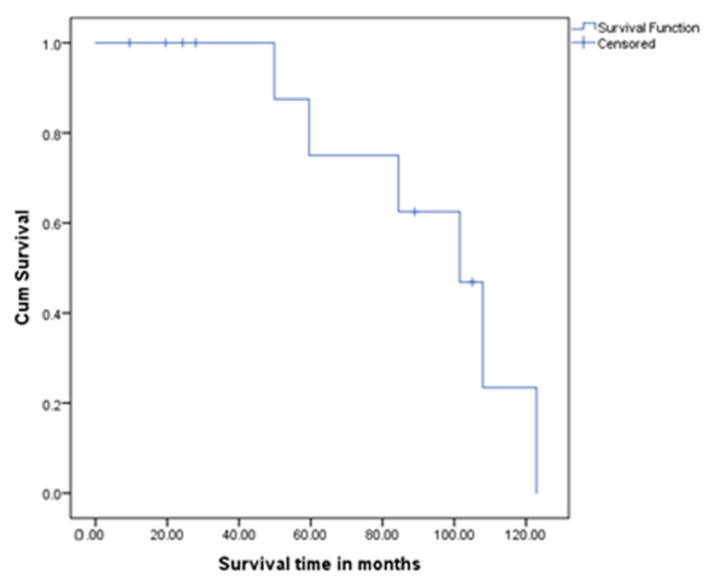
The Kaplan–Meier survival curve for the OS in cases with recurrent disease. The median overall survival (OS) was 94.2 months (95% CI: 74.4–114.0), and the 5-year OS was 33.3%. A lower Ki–67 index and low tumour grade (G1) correlated with improved survival (*p* = 0.011 and 0.006, respectively), whereas advanced T-stage (T2–3) was more frequent among deceased patients, although the small sample size limited statistical power.

Among the patients who developed recurrence, the median overall survival (OS) was 94.2 months (95% CI: 74.4–114.0), and the 5-year OS was 33.3%. A lower Ki–67 index and low tumour grade (G1) correlated with improved survival (*p* = 0.011 and 0.006, respectively), whereas advanced T-stage (T2–3) was more frequent among deceased patients, although the small sample size limited statistical power (Figure 4).

In patients with early recurrence (≤5 years post-diagnosis), the median OS was significantly lower at 59 months (*p* < 0.001, log-rank test). In patients with late recurrence (>5 years post-diagnosis), the median OS was not reached, with all three survivors exceeding 8.2 years (range: 8.2–14.2 years).

Patients with late recurrence (>5 years) had no mortality, compared to 77.8% mortality in early recurrence (*p* = 0.02, Fisher’s exact test). Late recurrence was associated with favourable tumour biology (Ki–67 < 0.25, *p* = 0.01) and T1 lesions (*p* = 0.04). In contrast, early recurrence was associated with higher Ki–67 (>5%, *p* = 0.008), advanced T-stage (T2–3, *p* = 0.04) and the presence of VHL (*p* = 0.03). The Charlson Comorbidity Index (CCI) did not differ between groups (*p* = 0.89), highlighting tumour biology as the dominant prognostic factor.

## 5. Discussion

In the present study, the 5-year disease-free and overall survival rates following pancreatic resection for PNETs were 147.0 and 143.3, respectively, highlighting an excellent survival outcome. In addition, the postoperative morbidity and mortality rates observed were low. Other studies have also found similar results [9,13]. Unlike pancreatic ductal adenocarcinoma, which carries a poor prognosis, PNETs generally have a more favourable clinical course, even in the presence of metastatic disease [14,15]. More recently, the incidental diagnosis of PNETs has increased up to sevenfold due to the widespread use of advanced imaging techniques [16]. Furthermore, the size of these lesions at diagnosis has considerably decreased, with tumours  <2 cm ranging from 26% to 61% [17,18,19]. With this increased incidence, more PNETs patients are being subjected to further surveillance and surgical resection, as these tumours are being diagnosed at an early resectable stage. In the present cohort, most tumours were small and solitary at presentation and located at the tail or body of the pancreas, resulting in patients undergoing a distal pancreatectomy. Histopathological analysis showed that most tumours were classified as low grade (G1, Ki–67 <3%), with an absence of perineural and vascular invasion. These findings are consistent with previously published data reflecting the less aggressive behaviour of PNETs [12,14].

Non-functioning PNETs comprise the majority of all PNETs, with 5-year overall survival rates of up to 58% being reported [6,20]. Recent studies have shown that this subtype of PNETs have a range of malignant potential, some of which can be locally invasive and metastasise early [21,22]. As these tumours do not secrete hormones, the majority of these tumours are found incidentally and may therefore present with advanced disease [20]. In tandem with the observations by Tan et al. [23], non-functioning tumours were more common than functioning tumours in the present study.

In contrast, functioning PNETs typically present earlier due to hormone-mediated symptoms. Although functioning tumours in our cohort demonstrated numerically higher 5-year survival rates, this finding lacked statistical significance. The recurrence rates were comparable between the two groups, suggesting that functional status alone is an unreliable prognostic marker.

Notably, the margin status did not have an impact on the OS. This aligns with a large US Neuroendocrine Tumour Study Group analysis (*n* = 854) which showed no survival difference between R0 and R1 resections (HR 1.16, *p* = 0.48) [23]. In contrast to PDAC, where margin status significantly affects 5-year survival [24], this suggests that tumour biology in PNETs may outweigh margin clearance in prognostic relevance.

The CCI emerged as an independent predictor of survival, with 40% of deaths attributed to non-oncologic causes. This mirrors SEER–Medicare data showing that comorbidities contribute to 38% of mortality in PNET patients [25].

The prediction of outcomes was further refined by tumour grade and Ki–67 index, both of which strongly predicted overall and disease-free survival and validated their centrality in the WHO classification system [26]. Although margin status, nodal involvement and vascular invasion did not independently influence survival, their significant association with recurrence supports their utility in guiding surveillance protocols, as recommended by ENETS guidelines [27].

Despite curative-intent resection, a subset of patients developed recurrence, primarily through hematogenous spread—a pattern well-documented in the ENETS study [27]. Our analysis identified male sex, vascular invasion, high tumour grade (G3), elevated Ki–67 index (>3%) and nodal metastases as independent predictors of recurrence. These findings are consistent with Yang et al.’s WHO-based classification study [6] and Zhang et al.’s validation of vascular invasion and lymph node metastasis as key prognostic factors. [24]. Notably, although perineural invasion was frequently observed, this lacked independent prognostic significance—a finding consistent with previous risk models [7,28].

The management of recurrent disease was largely multimodal, with the majority receiving systemic therapy, often with targeted agents such as everolimus or sunitinib. A subset underwent surgical resection, including isolated liver lesions and local recurrences, both of which yielded favourable long-term outcomes. These data support the role of aggressive local treatment in selected cases of oligometastatic or locoregional recurrence [29,30].

Consistent with previous reports [31,32], our findings confirm that late recurrences can occur up to 10 years after the resection of PNETs. For patients with high-risk pathological features (G3 differentiation, nodal metastases, or vascular invasion), the ENETS guidelines [27] advocate an intensified surveillance regimen; cross-sectional imaging (CT or MRI) every 6 months for the first 3 years, transitioning to annual studies thereafter. While this approach maximises the early detection of recurrent disease, its implementation requires a careful consideration of institutional resources and patient compliance, particularly for lifelong monitoring. Conversely, low-risk patients (G1, N0) could be safely monitored with less frequent imaging, in line with studies reporting <2% recurrence beyond five years with annual follow-up [17].

## 6. Conclusions

Curative-intent pancreatic resection for PNETs leads to favourable long-term outcomes with durable disease control, though tumour biology remains the dominant determinant of prognosis. High-grade histology (G3), larger tumours, vascular invasion and nodal metastases are significantly associated with poorer survival, while margin status shows limited impact, underscoring the primacy of intrinsic tumour characteristics over surgical technical factors. These findings justify risk-stratified surveillance, with high-risk patients warranting intensive imaging (CT/MRI every 6–12 months for 3–5 years) and extended follow-ups beyond five years due to late recurrence potential, whereas low-risk patients (G1, N0) may undergo less-frequent monitoring. Future paradigms should integrate clinicopathological factors with emerging molecular markers to optimise personalised management strategies for this biologically heterogeneous disease.

## Figures and Tables

**Table 1:** Different demographics and pathological features.

Clinical and Pathological Factors	Number of Patients	Percentage
Mean Age (years)	61.5	
Male/Female	1: 1	
Site of Tumour		
Head of Pancreas	15	17.40
Neck of Pancreas	1	1.20
Body of Pancreas	11	12.80
Body and Tail	9	10.50
Tail of Pancreas	49	57.00
Uncinate Process	1	1.20
Functionality		
Not Functioning	62	72.09
Functioning	24	27.91
Insulinoma	10	41.66
Carcinoid	5	20.83
Gastrinoma	1	4.17
Glucagonoma	7	29.17
VIPOMA	1	4.17
Associated Genetic Syndrome		
None	78	90.70
MEN 1	5	5.81
VHL	2	2.33
NF1	1	1.16
Type of Operation		
Distal Pancreatectomy	65	75.60
Pyloric Preserving Pancreaticoduodenectomy	5	5.80
Standard Whipple	13	15.10
Total Pancreatectomy	3	3.50
Number Of Tumours		
One	76	88.40
Two	4	4.70
Three	1	1.20
Multifocal	5	5.80
UICC (8th edition) TNM Staging System		
T (Tumour)		
T_1_	35	40.69
T_2_	30	34.88
T_3_	18	20.93
T_4_	3	3.48
N (Nodes)		
N_0_	66	76.74
N_1_	19	22.09
N_2_	1	1.16
M (Metastasis)		
M_0_	85	98.83
M_1_	1	1.16
Tumour Grade		
I	56	65.12
II	23	26.74
III	7	8.14
KI 67 Expression		
<3	56	65.12
3–20	23	26.74
>20	7	8.14
Perineural Invasion		
No	74	86.05
Yes	12	13.95
Vascular Invasion		
No	61	70.93
Yes	25	29.07
R Status (Resection Margin Status)		
0	67	77.90
1	19	22.10
Complications	32	37.2
Postoperative collection	7	8.1
Small bowel Ileus	6	6.9
Chest infection	4	4.7
Minor leak/pancreatic fistula	2	2.3
Pancreatico-jejunostomy (PJ) leak	2	2.3
Pancreatitis	2	2.3
Intraoperative bleeding	2	2.3
Chyle leak	1	1.2
Major 2ry postoperative bleeding	1	1.2
SMV thrombosis	1	1.2
Pleural effusion	1	1.2
Clavien–Dindo Classification		
2	26	81.3
3 A	5	15.6%
5	1	3.1
Mortality	15	17.44
Reason for Mortality		
Metastatic PNET	7	46.70
Metastatic breast cancer	2	13.30
Cardiovascular/medical disease	6	40.00

**Table 2:** Different demographics and pathological features of recurrent diseases.

Variables	Number of Patients	Percentage
**Type of surgery**		
Distal pancreatectomy	6	50
Standard pancreatoduodenectomy	3	25
Pylorus-preserving pancreatoduodenectomy	2	16.7
Total pancreatectomy	1	8.3
**Tumour focality**		
Unifocal	11	91.7
Multifocal	1	8.3
**UICC (8th edition) TNM staging system**		
**T (Tumour)**		
T_1_	1	8.3
T_2_	5	41.6
T_3_	4	33.3
T_4_	2	16.7
**N (Nodes)**		
N_0_	7	58.3
N_1_	5	41.6
N_2_	0	0
**M (Metastasis)**		
M_0_	5	41.6
M_1_	7	58.3

**Table 3:** Univariable and multivariable Cox regression analysis for DFS. The multivariate Cox regression analysis included factors that were statistically significant in univariate testing (*p* < 0.05) or clinically relevant to pancreatic tumour outcomes.

Variables	Univariate		Multivariate	
	*p* Value	HR (95th C.I.)	*p* Value	HR (95th C.I.)
Age	0.818	0.995 (0.958–1.035)		
Sex (M/F)	0.039	4.026 (1.07–15.147)	0.531	1.783 (0.293–10.864)
Size of tumour	0.009	1.013 (1.003–1.023)	0.007	1.017 (1.005–1.029)
Vascular invasion	0.003	6.353 (1.885–21.409)	0.27	2.715 (0.46–16.029)
Perineural invasion	0.042	3.526 (1.046–11.883)	0.114	3.688 (0.73–18.631)
R status	0.498	1.576 (0.423–5.872)		
CCI (Charlson Comorbidity Index)	0.001	1.63 (1.235–2.152)	0.031	1.618 (1.044–2.508)
KI 67 expression %	0.006	1.142 (1.039–1.256)	0.002	1.261 (1.087–1.463)
Function	0.113	0.185 (0.023–1.495)		
T (1–2/3–4)	0.158	2.298 (0.724–7.289)		
N (0/1 and 2)	0.015	4.527 (1.342–15.272)	0.778	0.725 (0.077–6.83)
Tumour grade 1 versus 2 and 3	0.006	5.653 (1.651–19.35)	0.826	1.275 (0.147–11.027)
Number (single/multiple)	0.561	0.544 (0.07–4.237)		

**Table 4:** Univariable and multivariable Cox regression analysis for OS (the multivariate Cox regression analysis included factors that were statistically significant in univariate testing (*p* < 0.05) or clinically relevant to pancreatic tumour outcomes).

Variables	Univariate		Multivariate	
	*p* Value	HR (95th C.I.)	*p* Value	HR (95th C.I.)
Age	0.055	1.041 (0.999–1.085)		
Sex (F//M)	0.624	1.289 (0.467–3.561)		
Size of Tumour	0.039	1.009 (1–1.018)	0.007	1.015 (1.004–1.026)
Vascular Invasion (no/yes)	0.097	2.363 (0.856–6.525)		
Perineural Invasion (no/yes)	0.677	1.374 (0.308–6.122)		
CCI (Charlson Comorbidity Index)	0.002	1.396 (1.128–1.728)	<0.001	2.116 (1.395–3.21)
KI 67 expression %	0.134	1.077 (0.977–1.187)	0.025	1.193 (1.022–1.394)
N (0/1 and 2)	0.198	0.464 (0.144–1.493)		
T (1–2/3–4)	0.425	1.549 (0.528–4.538)		
Tumour grade 1 versus 2 and 3	0.13	2.205 (0.793–6.128)	0.046	0.139 (0.02–0.968)
Number (single/multiple)	0.373	0.397 (0.052–3.024)		
R Status	0.254	1.953 (0.618–6.179)		
Function	0.216	0.39 (0.088–1.731)		

## Data Availability

The data presented in this study are available on request from the corresponding author due to patient confidentiality.
